# Effect of injection of botulinum toxin on decreasing the symptoms and signs of masticatory muscles in patients with temporomandibular dysfunction

**DOI:** 10.15171/joddd.2019.020

**Published:** 2019-08-14

**Authors:** Mohammad Ali Ghavimi, Javad Yazdani, Atena Afzalimehr, Arezoo Ghoreyshizadeh, Seyed Vahid Dehnad

**Affiliations:** ^1^Department of Oral and Maxillofacial Surgery, Faculty of Dentistry, Tabriz University of Medical Sciences, Tabriz, Iran; ^2^Department of Pediatric Dentistry, Faculty of Dentistry, Tabriz University of Medical Sciences, Tabriz, Iran

**Keywords:** Temporomandibular joint dysfunction, botulinum toxin, pain, articular click

## Abstract

***Background.*** Temporomandibular joint dysfunction (TMD) is a term that describes problems in the masticatory system, including the temporomandibular joint, the dento-muscular system and the supporting bones. Injection of botulinum toxin, as a noninvasive technique, might be useful in decreasing symptoms such as muscular spasm, dystonia, migraine headaches and TMD. Therefore, the aim of the present study was to evaluate the effect of injection of botulinum toxin on decreasing the symptoms and signs of masticatory muscles in patients with TMD.

***Methods.*** A total of 61 patients were consecutively included in the present study in 2016‒2017. All the subjects received a 50-unit injection of Dysport botulinum toxin in the masseter muscles using an extraoral injection technique. The results of the injection were evaluated in terms of pain severity using VAS, clinical evaluations of the joint click through palpation and by determining the inter-incisal distance. The patients underwent follow-up examinations at 1-week, 3-month and 6-month intervals after injection. Data were analyzed with appropriate statistical tests.

***Results.*** Comparison of pain severity and articular clicks at different intervals showed decreases in these parameters over time, with significant differences between the time intervals (P<0.05). Comparison of mouth opening at different intervals showed increases in mouth opening over time.

***Conclusion.*** The results of the present study showed that injection of botulinum toxin can be used in patients with TMD as a non-invasive treatment modality

## Introduction

Temporomandibular joint dysfunction (TMD) describes problems in the masticatory system, including TM joint, the dento-muscular system and the supporting bones. The prevalence of this disorder in the adults is around 40‒70% and in children in the deciduous dentition period the prevalence is approximately 16%, with 90% in the mixed dentition period.^1‒3^ In the majority of cases, TMD is associated with pain and jaw dysfunction; however, it might be manifested in the form of headaches, cervical pain and facial swelling. The clinical manifestations of TMD are very similar to those of chronic low back pain.^4^ Conventionally, to treat TMD, first noninvasive techniques, including medications (analgesics, narcotics, anti-inflammatory agents and muscle relaxants) are used, followed by physiotherapy. If the symptoms persist, the second line of treatment includes irrigation of the joint and invasive surgeries to replace the joint, etc. Since great morbidity is associated with surgical procedures, it is necessary to apply all the noninvasive treatment modalities to decrease the symptoms and signs of TMD. None of these treatment modalities are completely effective and even some are associated with untoward complications.^5^ Therefore, researchers are trying to find novel treatment modalities for TMD.

Botulinum toxin is a neurotoxin derived from *Clostridium botulinum*. It results in the paralysis of nerves by blocking the release of acetylcholine.^6^ The toxin is a known treatment to remove skin wrinkles and some studies have evaluated the use of this toxin to relieve muscular spasm, dystonia, migraine headaches, blepharospasm, strabismus, spasmodic torticollis, removal of cleft lip scars and the capability to permanently accept botulinum toxin.^7^ Some studies have reported improvements in the anatomic position of TMJ subsequent to the injection of the toxin into the lateral pterygoid muscles; in addition, the results have shown that the toxin can improve the clinical status of TMJ.^8,9^ In addition, a study evaluated the success of the injection of this toxin in the treatment of myofascial pains, including TMD. Studies have shown improvements in pain severity and patients’ psychological status.^10^ Furthermore, several studies have evaluated the treatment of TMJ dislocations, including the habitual neurologic cases. In a case report by Vazquez et al,^11^ infiltration of botulinum toxin into the muscle involved resulted in the successful management of the disorder in a conservative manner. However, Ernberg et al^12^ evaluated the effect of injection of this toxin into the masseter muscle on relieving TMD symptoms and reported a significant difference between the group receiving normal saline solution and the group receiving botulinum toxin. In a review study, Ihde et al^13^ reported that use of botulinum toxin was effective in treating myofascial pains, including that of TMD.

There is a limited number of studies available on the use of botulinum toxin for the treatment of myofascial pains; in addition, these studies have not elaborated on significant improvements in these conditions. The results of these studies have raised many questions in relation to the role of masticatory muscles in myofascial pain. If muscular problems are accepted as a chief etiologic factor for TMD, only muscular function should be evaluated as a serious etiologic factor for myofascial pains. Therefore, the aim of the present study was to evaluate the effect of injection of botulinum toxin on decreasing the symptoms and signs of masticatory muscles in patients with TMD.

## Methods

In the present clinical trial, 61 patients with TMD were selected consecutively during 2016‒2017 and included in the study based on inclusion and exclusion criteria after signing informed consent forms. All the subjected completed the study procedures with no dropouts. The inclusion criteria consisted of an age range of 18‒50 years and signing an informed consent form.

The exclusion criteria consisted of systemic conditions, a history of surgery or trauma in the TMJ area, a history of injection of botulinum toxin in the TMJ area, and a history of physiotherapy with heat in the TMJ area.

The diagnoses were based on medical history, an increase in muscular tonicity and muscular spasm, including pain triggers. Therefore, all the patients with a diagnosis of TMD, complaining of pain in masticatory muscles related to TMD were selected. The patients’ severity of pain was recorded based on VAS. To this end, each patient was asked to select a number from zero to ten based on the severity of pain. The presence or absence of articular clicks (unilateral or bilateral) was evaluated and recorded by palpating the TMD area during the function of the mandible. The presence or absence of limitation in mouth opening was evaluated and recorded by measuring the inter-incisal distance. All the examinations were carried out by two oral and maxillofacial surgeons who were blinded to the aims of the study (a double-blind design). All the patients underwent a conservative treatment protocol (use of a soft diet, muscular relaxation, refraining from excessive opening of the mouth, use of local or systemic nonsteroidal anti-inflammatory drugs) for 3 months before the interventions in the study. Then 50 units of Dysport botulinum toxin (ISPEN, UK) were injected into the masseter muscle in each patient. An extraoral technique was used in the bulk of the masseter muscle to deposit the toxin at a depth of 1 cm at 3 points. In cases in which the symptoms and signs were bilateral, the injection was carried out bilaterally; otherwise, unilateral injection was carried out. All the patients were followed at 1-week, 1-month, 3-month and 6-month intervals after the injection. VAS was used to evaluate the presence or absence articular clicks by palpation and the amount of mouth opening by measuring the inter-incisal distance during each follow-up visits. All the patients were recommended to continue conservative treatment modalities and refer to the clinic if the symptoms and signs recurred.

The protocol of the study was approved by the Ethics Committee of Tabriz University of Medical Sciences under the code TBZMED.REC.1396.1098. No costs were inflicted on the patients. All the patients underwent routine procedures of diagnosis and treatment and were free to leave the study whenever they wished without stating any reasons. Data were analyzed with descriptive statistics (frequencies, percentages, means and standard deviations) using repeated measures ANOVA and Friedman test with the use of SPSS 20. Statistical significance was set at P<0.05.

## Results

A total of 33 female subjects (54.1%) and 28 male subjects (45.9%) were included in the present study. The mean age of the subjects was 31.86±6.82 years and 21 (42.6%) and 35 (57.4%) subjects had bilateral and unilateral TMD, respectively.

Table 1 presents the descriptive statistics of pain severities before injection and 1 week, 1 month, 3 months and 6 months after injection of the toxin.

**Figure 1:**
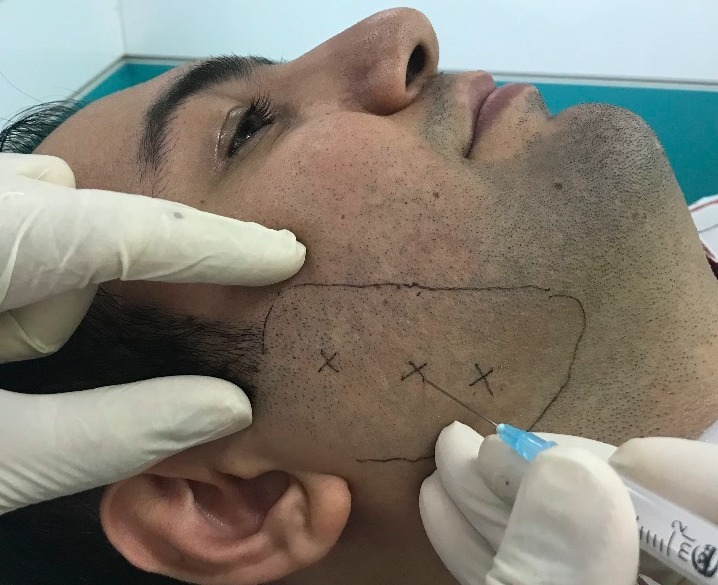
Injecting into the bulk of the masseter muscle to deposit the toxin at a depth of 1 cm at 3 points.

**Table 1:** Descriptive statistics of pin severity

**Pain/interval**	**1**	**2**	**3**	**4**	**5**	**6**	**7**
**Before injection**	0	9 (14.8%)	23 (37.7%)	12 (19.7%)	13 (21.3%)	3 (4.9%)	1 (1.6%)
**One week after injection**	9 (14.8%)	17 (27.9%)	27 (44.3%)	7 (11.5%)	1 (1.6%)	0	0
**One month after injection**	18 (29.5%)	24 (39.3%)	15 (24.6%)	3 (4.9%)	1 (1.6%)	0	0
**Three months after injection**	27 (44.3%)	25 (41%)	7 (11.5%)	4 (2%)	0	0	0
**Six months after injection**	42 (68.9%)	12 (19.7%)	7 (11.5%)	0	0	0	0

As shown in the Table, before injection 23 subjects (37.7%) (the highest frequency) had a pain severity of 3 and one (1.6%) subject (the lowest frequency) had a pain score of 7 and no subject had a pain score of 1.

One week after injection, 27 subjects (44.3%) (the highest frequency) still had a pain score of 3 and no subject had a score of 6 or 7. One month after injection, the severity of pain decreased and 24 subjects (39.3%) had a pain score of 2. At this interval again, no subjects had a pain score of 6 or 7. Three months after injection, 27 subjects (44.3%) (the highest frequency) had a pain score of 1 and no subject had a pain score of 5‒7. Six months after injection, 42 subjects (68.9%) (the majority of the subjects) had a pain score of 1 and the subjects had pain severity up to score 3. The differences in pain severities of the objects at different time intervals after injection were significant based on the results of Friedman test (P<0.001).

Table 2 presents the results of evaluations in relation to articular clicks. As shown in the Table, before injection of the toxin 45 subjects (73.8%) had articular clicking, which decreased to 25 subjects (41%) at 1-week interval after injection. One month after injection, 15 subjects (24.6%) had articular clicking, which decreased to 9 subjects (14.8%) 3 months after injection. Six months after injection, only 6 subjects (9.8%) exhibited articular clicking. Friedman test revealed significant differences in articular clicking between the different time intervals after injection of the toxin (P<0.001).

**Table 2:** Descriptive statistic of articular clicks

**Pain/interval**	**Without**	**With**
**Before injection**	16 (26.2%)	45 (73.8%)
**One week after injection**	36 (59%)	25 (41%)
**One month after injection**	46 (75.4%)	15 (24.6%)
**Three months after injection**	52 (85.2%)	9 (14.8%)
**Six months after injection**	55 (90.2%)	6 (9.8%)

Normal distribution of data was evaluated with the use of Kolmogorov-Smirnov test in order to compare the extent of mouth opening at different time intervals before and after injection of the toxin. The results of the test showed that data were not distributed normally only at 1-week and 3-month intervals after injection of the toxin (P>0.05).

The mean mouth opening was determined at different time intervals and the results are presented in Table 3. As shown in the table, there was a difference of 10.8 mm in mouth opening before injection and 6 months after injection. Repeated measures ANOVA was used to analyze such difference.

**Table 3:** Descriptive statistics of the extent of mouth opening

	**Evaluation interval**	**Mean ± SD**
**Mouth opening**	**Before injection**	31.29±6.8
	**One week after injection**	36.09±4.8
	**One month after injection**	38.27±4.37
	**Three months after injection**	40.67±3.7
	**Six months after injection**	42.09±3.7

## Discussion

TMD-related disorders are usually due to pain in the joints and facial muscles. In relation to arthralgia, there is inflammation in tissues in association with limitations in movements; in addition, there is pain at loading and lack of reflexes during mastication and pain on mouth opening. However, the origin of chronic pain in the facial muscles is still to be elucidated. Furthermore, it is still unknown whether chronic pain in the facial muscles in TMD patients has a relationship with other similar conditions such as fibromyalgia.^9,14^ Muscle relaxation might be lost under the influence of various factors, including inadequate concentration of active toxin adjacent to motor end plate, the presence of antibodies against BTX-A, improper regeneration and inadequate medication pool.^15^

The results of the present study and the majority of other studies indicated a higher prevalence of TMD in female patients. Most researchers believe that the difference in gender distribution is due to the higher frequency of medical visits by female subjects. In addition, some researchers believe that it is due to the lower pain tolerance by women. In addition, a study has shown that a higher rate of referral by female subjects to receive treatment for TMD is directly related to sex hormone levels and estrogen.^16‒18^ In addition, comparison of pain and articular clicking at different intervals in the present study showed that these variables decreased over time. However, comparison of the extent of mouth opening at different intervals showed an increase in mouth opening over time. In this context, in relation to the severity of pain, approximately 69% of the patients had the least pain severity (a pain score of 1) 6 months after injection and only one subject had a pain score of 3. In addition, 6 months after injection only 6 subjects (9.8%) exhibited articular clicking. A study evaluated the effect of injecting botulinum toxin on resolving TMJ disorders; the results showed improvements in pain severity and the performance of TMJ, consistent with the results of the present study. The study above, too, did not have a control group; however, the follow-up period was shorter and evaluations were carried out before injection and up to 8 weeks after injection.^9^ In addition, in another study the success of injection of botulinum toxin was evaluated in the treatment of myofascial pains due to TMD. The results showed improvements in pain severity and psychological conditions of patients, consistent with the results of the present study. However, the study had some limitations such as short follow-up period of patients, small sample size and no evaluation of the anatomic status of the articular disk.^10^

In addition, some studies have evaluated the treatment of dislocation of TMJ in habitual neurologic cases. In a case report by Vazquez et al,^11^ infiltration of botulinum toxin into the muscle involved resulted in conservative management of the condition. Some studies have reported improvements in the anatomic position of TMJ after injection of botulinum toxin into the lateral pterygoid muscle, with the results showing that the injection of the toxin might improve the clinical status of TMJ. The results of the study above, too, were consistent with those of the present study; however, the study had some limitations, including short follow-up period of the subjects, small sample size and lack of a control group.^8^

Ernberg et al^12^ evaluated the effect of injection of botulinum toxin into the masseter muscle on decreasing the symptoms and signs of TMD. The results showed no significant differences between the two groups, with one receiving normal saline solution and the other one receiving botulinum toxin, which is different from the results of the present study. It should be noted that the study above had a small sample size and with such a small sample size it is not possible to rule out the effect of injection of botulinum toxin. In addition, in the study above samples with TMD were not differentiated from others with other myofascial pains. Therefore, inconstancy in the results is possible.

Ihde et al^13^ reported that use of botulinum toxin was effective in the treatment of myofascial pains due to TMD. The results of this study were similar to the present study in one-month follow-up. However, in one-week follow-up in the present study, pain decrease was lower compared to the study above.

## Conclusion

It might be concluded from the results of the present study that injection of botulinum toxin can be used as an adjunct for the noninvasive treatment of TMD-related disorders. In addition, based on the results of the present study, it is expected that the best results would be achieved in relation to a decrease in pain severity and articular clicking 6 months after injection of the toxin.

## Authors’ contributions

MAG was responsible for the concept and design of the work, data collection, data analysis and interpretation, drafting the article, critical revision of the article and final approval of the version to be published. JY was responsible for data collection and final approval of the version to be published. AA contributed to data collection, data analysis and interpretation, drafting the article and critical revision of the article. AG contributed to drafting the article, critical revision of the article and final approval of the version to be published. SVD contributed to data analysis and interpretation, drafting the article and critical revision of the article.

## Acknowledgments

None

## Funding

Not applicable.

## Competing Interests

The authors declare no competing interests with regards to the authorship and/or‏ publication of this article.

## Ethics Approval

The protocol of the study was approved by the Ethics Committee of Tabriz University of Medical Sciences under the code TBZMED.REC.1396.1098.
